# Intratumoral Combinatorial Administration of CD1c (BDCA-1)^+^ Myeloid Dendritic Cells Plus Ipilimumab and Avelumab in Combination with Intravenous Low-Dose Nivolumab in Patients with Advanced Solid Tumors: A Phase IB Clinical Trial

**DOI:** 10.3390/vaccines8040670

**Published:** 2020-11-10

**Authors:** Julia Katharina Schwarze, Gil Awada, Louise Cras, Jens Tijtgat, Ramses Forsyth, Inès Dufait, Sandra Tuyaerts, Ivan Van Riet, Bart Neyns

**Affiliations:** 1 Department of Medical Oncology, Universitair Ziekenhuis Brussel, Laarbeeklaan 101, 1090 Brussels, Belgium; gil.awada@uzbrussel.be (G.A.); jens.tijtgat@uzbrussel.be (J.T.); ines.dufait@uzbrussel.be (I.D.); sandra.tuyaerts@uzbrussel.be (S.T.); bart.neyns@uzbrussel.be (B.N.); 2 Department of Anatomopathology, Universitair Ziekenhuis Brussel, Laarbeeklaan 101, 1090 Brussels, Belgium; louise.cras@uzbrussel.be (L.C.); ramses.forsyth@uzbrussel.be (R.F.); 3 Stem Cell Laboratory, Department of Hematology, Universitair Ziekenhuis Brussel, Laarbeeklaan 101, 1090 Brussels, Belgium; ivan.vanriet@uzbrussel.be

**Keywords:** cancer immunotherapy, dendritic cells, intratumoral, myeloid dendritic cells, conventional dendritic cells, immune checkpoint inhibitors, tumor microenvironment

## Abstract

Intratumoral (IT) myeloid dendritic cells (myDCs) play a pivotal role in re-licensing antitumor cytotoxic T lymphocytes. IT injection of the IgG_1_ monoclonal antibodies ipilimumab and avelumab may induce antibody-dependent cellular cytotoxicity, thereby enhancing the release of tumor antigens that can be captured and processed by CD1c (BDCA-1)^+^ myDCs. Patients with advanced solid tumors after standard care were eligible for IT injections of ≥1 lesion with ipilimumab (10 mg) and avelumab (40 mg) and intravenous (IV) nivolumab (10 mg) on day 1, followed by IT injection of autologous CD1c (BDCA-1)^+^ myDCs on day 2. IT/IV administration of ipilimumab, avelumab, and nivolumab was repeated bi-weekly. Primary objectives were safety and feasibility. Nine patients were treated with a median of 21 × 10^6^ CD1c (BDCA-1)^+^ myDCs, and a median of 4 IT/IV administrations of ipilimumab, avelumab, and nivolumab. The treatment was safe with mainly injection-site reactions, but also immune-related pneumonitis (*n* = 2), colitis (*n* = 1), and bullous pemphigoid (*n* = 1). The best response was a durable partial response in a patient with stage IV melanoma who previously progressed on checkpoint inhibitors. Our combinatorial therapeutic approach, including IT injection of CD1c (BDCA-1)^+^ myDCs, is feasible and safe, and it resulted in encouraging signs of antitumor activity in patients with advanced solid tumors.

## 1. Introduction

Remarkable antitumoral activity has been achieved in various cancer types by blocking the inhibitory T-cell receptor cytotoxic T-lymphocyte-associated protein 4 (CTLA-4) or the programmed cell death protein 1 (PD-1/PD-L1) axis [1]. Anti-CTLA-4 (e.g., ipilimumab) and anti-PD-1 (e.g., pembrolizumab, nivolumab) monoclonal antibodies, both as monotherapy and in combination regimens, have become a standard of care treatment option in patients with melanoma, renal cell carcinoma, non-small cell lung cancer, urothelial cancer, and Hodgkin lymphoma. The indications for immune checkpoint inhibitors are continuously expanding [2,3,4,5,6,7,8,9,10]. Despite the impressive improvement in survival that was obtained with immune checkpoint inhibition, most advanced cancer patients will need additional treatment options, as only a minority of patients remains disease-free five years after initiating therapy.

The concept of the “cancer-immunity cycle” introduced by Chen and Mellman refers to the potential of a patient’s immune system to recognize cancer cells and mount an adaptive antitumor immune response [11,12,13,14,15]. A pivotal role in initiating antigen-specific antitumoral immunity has recently been attributed to specific types of myeloid dendritic cells (myDCs) [14,16]. It has been shown in mouse models that myDCs are essential for priming antitumor T-cell responses, with type 1 conventional dendritic cells (cDC1), being characterized by the expression of CD103 and dependent on the transcription factor Batf3, mediating CD8^+^ cytotoxic T lymphocytes (CTL), and type 2 conventional dendritic cells (cDC2) mediating CD4^+^ T-cell responses against tumor cells [17]. In order to properly prime antitumoral T-cells, myDCs need to (1) process tumor antigens, (2) undergo maturation in order to induce upregulation of CCR7, (3) migrate to tumor-draining lymph nodes, and (4) present tumor antigens to T cells in these secondary lymphoid organs [15]. In addition, myDCs are essential in “re-licensing” antitumor T lymphocytes to eradicate tumor cells within the tumor microenvironment (TME) [14]. Moreover, animal models indicate that the exclusion of myDCs from the TME is a tumor-intrinsic mechanism of immune evasion. Amongst other mechanisms, activation of the oncogenic WNT/β-catenin pathway can lead to the exclusion of Batf3-expressing myDCs from the TME by downregulating the production of chemokines necessary to attract myDCs from the blood to enter the TME [16,18,19]. The absence of myDCs at the invasive margin and within metastases has been correlated with defective CTL activation allowing for metastases to escape the antitumoral immune response [20]. Additionally, an analysis of data from The Cancer Genome Atlas (TGCA) indicates a correlation between the presence of specific types of myDCs and improved survival in various tumor types [14,15,21]. The presence of myDCs was more strongly correlated with T-cell infiltration into tumors as compared to the neoantigen load in 266 melanomas from TCGA [22].

Myeloid DCs are circulating in the peripheral blood and are classified according to their surface markers and function [23,24]. The isolation of myDCs from peripheral blood mononuclear cells (PBMC) by immunomagnetic beads has become possible for CD1c (BDCA-1)^+^ myDCs (cDC2), and BDCA-4^+^ plasmacytoid dendritic cells (pDCs) [25]. Notably, human CD1c (BDCA-1)^+^ myDCs are heterogeneous, with a CD14-positive subpopulation that is immunosuppressive and a CD14-negative subpopulation that is capable of mediating antitumor immune responses induced by immunogenic cancer cell death [26,27,28].

When properly activated, human CD1c (BDCA-1)^+^ myDCs secrete high levels of interleukin-12 (IL-12) and potently prime CTL responses [29]. In vitro, IL-12 production by CD1c (BDCA-1)^+^ myDCs can be boosted by exogenous interferon-gamma (IFN-γ) [29]. Optimal maturation with secretion of IL-12 as well as the orientation of stimulated T lymphocytes towards a Th1 phenotype is only achieved following Toll-like receptor stimulation [30]. 

The therapeutic potential of cellular vaccines that contain antigen-loaded CD1c (BDCA-1)^+^ myDCs, also in combination with pDCs, has already been under investigation in early clinical trials in patients with metastatic melanoma or prostate cancer indicating objective tumor responses and immunogenicity [31,32,33]. Recently, it has been shown in mouse models that successful anti-PD-1 checkpoint blockade requires the crosstalk between T cells and DCs involving the cytokines IFN-γ and IL-12, and that especially cDC1 are required [34].

Previously, intratumoral delivery of the anti-CTLA-4 monoclonal antibody ipilimumab had shown equivalent antitumoral activity when compared to systemic administration in mouse models with better tolerance [35,36]. In addition, intratumoral administration of ipilimumab in combination with interleukin-2 (IL-2) was investigated in a phase I trial in patients with unresectable stage III/IV melanoma, where a local response of injected lesions was observed in 67% patients, and an abscopal response in 89% [37].

Following intratumoral injection, high local concentrations of ipilimumab and avelumab will efficiently block their targets in the TME. In addition, these IgG_1_ monoclonal antibodies can elicit antibody-dependent cellular cytotoxicity (ADCC) and complement-dependent cytotoxicity (CDC) against PD-L1-expressing tumor cells and CTLA-4-expressing regulatory T cells, thereby enhancing the release of tumor antigens and reducing immunosuppression in the TME [38,39,40]. The released tumor-associated antigens and damage-associated molecular pattens will favor antigen uptake and the maturation of intratumoral co-administered CD1c (BDCA-1)^+^ myDCs. After migration to the tumor-draining lymph nodes, the myDC will then present tumor-associated antigens to naïve T cells, thereby inducing adaptive immunity. It has been demonstrated that normal immune cell subsets are an unlikely target for avelumab mediated toxicity; therefore, CD1c (BDCA-1)^+^ myDCs that upregulate PD-L1 are not expected to become neutralized upon exposure to avelumab [39].

In this phase IB clinical trial, we investigate the safety and feasibility of a combined immunotherapeutic approach that includes the intralesional administration of autologous, non-substantially manipulated CD1c (BDCA-1)^+^ myDCs plus ipilimumab and avelumab in combination with intravenous low-dose nivolumab in patients with advanced solid tumors who progressed on standard of care treatment options.

## 2. Materials and Methods

### 2.1. Patient Eligibility and Regulatory Approval

The patients with histologically advanced solid tumors who progressed on available standard of care systemic treatment options, and who had metastatic disease amenable for intratumoral injection (non-visceral lesions) were eligible for participation in this trial. Other key inclusion criteria included: age ≥18 years; Eastern Cooperative Oncology Group (ECOG) performance status of ≤1; adequate organ function within 14 days prior to enrollment; and, negative serologic tests for human immunodeficiency virus (HIV), syphilis, hepatitis B, and hepatitis C. The subjects were excluded when they had leptomeningeal metastases, untreated, or symptomatic metastases of the central nervous system, need for systemic corticosteroids, and a history of autoimmune diseases. The institutional medical ethics committee of Universitair Ziekenhuis Brussel and the Belgian Federal Agency for Medicines and Health Products approved this clinical trial (ClinicalTrials.gov identifier: NCT03707808). All of the patients provided written informed consent.

### 2.2. Study Design and Treatment

This is an open-label, single-center, phase I clinical trial. On day 1 of the treatment schedule, patients underwent a leukapheresis for isolation of PBMC. Subsequently, avelumab (Bavencio^®^, Pfizer, 200 mg/10 mL solution) was administered by intratumoral injection of a maximum total dose of 40 mg (= 2 mL of a 200 mg/10 mL solution), followed by intratumoral injection of ipilimumab (Yervoy^®^, Bristol–Myers Squibb (BMS), 50 mg/10 mL solution) at a maximum total dose of 10 mg (= 2 mL of a 50 mg/10 mL solution). Nivolumab (Opdivo^®^, BMS) was administered by a 15 min intravenous infusion at a fixed dose of 10 mg. On day 2 of the treatment schedule, the isolated CD1c (BDCA-1)^+^ myDCs were intratumorally injected in the same lesions as on day 1. The patients were treated with the total amount of isolated autologous, non-substantially manipulated CD1c (BDCA-1)^+^ myDCs. The injected volume per lesion ranged from 0.1 mL for lesions <0.5 cm to 4.0 mL for lesions >5 cm in longest diameter. Intratumoral injections of avelumab and ipilimumab were repeated bi-weekly (q2w), when possible, as well as intravenous administration of nivolumab. Study treatment with nivolumab, ipilimumab, and avelumab was discontinued in the case of progressive disease according to the iRECIST criteria, unacceptable adverse events, disappearance of injectable lesions, or patient withdrawal.

### 2.3. Leukapheresis and Isolation of CD1c (BDCA-1)^+^ MyDCs

**Figure A1:**
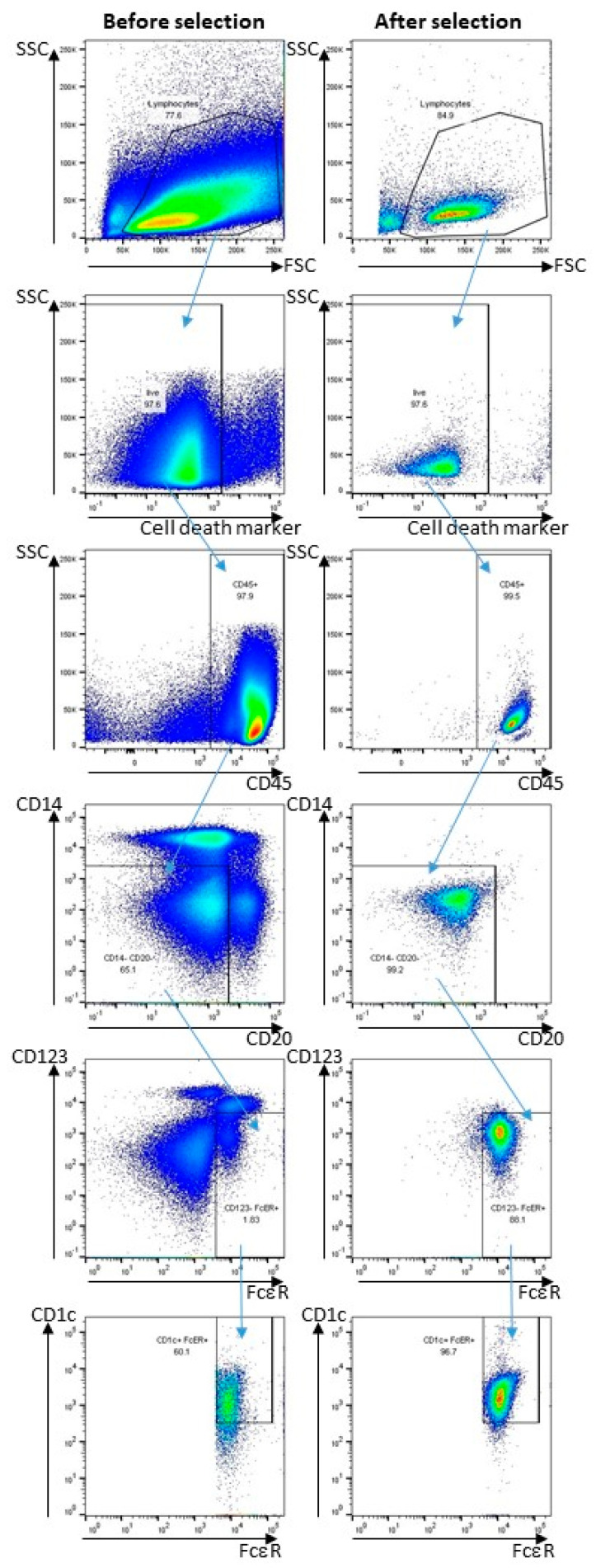
Gating strategy and example of flow cytometric analysis of CD1c (BDCA-1)^+^/CD14^-^myDCs.

PBMC were obtained by leukapheresis of 15 L of blood and, next, CD14^+^ and CD19^+^ cells were depleted, followed by positive selection of CD1c (BDCA-1)^+^ myDCs while using the CD1c (BDCA-1)^+^ Dendritic Cell Isolation Kit (Miltenyi Biotec, Bergisch Gladbach, Germany) on the immunomagnetic CliniMACS^®^ Plus isolation system (Miltenyi Biotec). The isolated fraction was concentrated by centrifugation and then resuspended in phosphate-buffered saline (PBS)/ethylenediaminetetraacetic acid (EDTA) (Miltenyi Biotec) containing 0.5% human albumin to obtain a cell suspension at the concentration (cells/mL) desired for clinical administration. Purity of the isolated CD1c (BDCA-1)^+^ myDC cell fraction was analyzed by flow cytometry (MACS Quant Analyzer 10, Miltenyi Biotec) while using the following monoclonal antibodies: CD14 PE, CD45 APC-Vio700, CD20 PE-Vio770, CD123 APC, FcεR VioBlue, and CD1c FITC with PI for dead cell exclusion (all antibodies from Miltenyi Biotec). CD1c (BDCA-1)^+^ myDCs were gated, as follows: the cells were first gated based on FSC/SSC characteristics, followed by exclusion of dead cells. Next, CD45^+^ were gated, followed by the exclusion of CD14^-^ CD19^-^ cells. On this gate, CD123^-^ FcεR^+^ CD1c^+^ cells were identified as CD1c (BDCA-1)^+^ myDCs. Figure A1 shows a representative figure showing the gating strategy. The predefined release criteria for the cell product were a viability of >50% and a purity of >85%.

### 2.4. Assessment of Tumor Response and Toxicity

Tumor assessment was performed by whole-body ^18F^FDG-PET/CT at baseline and every 12 weeks thereafter. The objective response rates were evaluated while using the modified Response Evaluation Criteria in Solid Tumors for immunotherapy (iRECIST). Safety assessments were made on a continuous base throughout the treatment phase by ways of clinical examination and blood analysis as well as up to 30 days after the last administration. Adverse events were cataloged and graded according to the Common Terminology Criteria for Adverse Events version 5.0 (CTCAEv5.0).

### 2.5. Tumor Biopsies and Tissue Analysis

Repetitive biopsies or fine needle aspirations of injected lesions were performed before the injection of study medication, when feasible. Biopsies were obtained while using an 18G Vacu-Cut^®^ needle (BD BARD^®^). Hematoxylin and eosin (H&E) staining and immunohistochemistry for SOX-10, CD3, CD8 and PD-L1 were performed on formalin-fixed, paraffin-embedded (FFPE) tissue blocks. CD3 2GV6 Ventana (Roche, Basel, Switzerland), CD8 SP57 (Roche), SOX10 SP267 Cell Marque (Roche), and PD-L1 22c3 (Agilent, CA, USA) antibodies were used. The evaluation for immunoreactivity was performed by a pathologist according to a semi-quantitative scoring system. The Panoramic SCAN II BF was used for scanning representative tissue slides.

On biopsies of interest, we performed multiplexed immunofluorescence with the UltiMapper™ I/O APC kit, containing antibody-conjugates against CD11c, CD20, CD68/CD163, and MHC Class II, and the UltiMapper™ I/O PD-L1 kit (Ultivue, Cambridge, MA, USA), containing antibody-conjugates against CD8, CD68, PD-L1, and panCK/SOX10. DAPI was used for nuclear counterstain. Staining was conducted on a Leica Biosystems BOND RX autostainer. Multiplex image acquisition was achieved while using the Zeiss Axio Scan.Z1 slide scanner. The images were analyzed using HALO 3.0 software (Indica Labs, Albuquerque, NM, USA). The same presets were used for all of the biopsies. A tissue sample of a tonsil was used as a positive control.

### 2.6. Statistical Analysis

Descriptive summary statistics are provided for demographics, safety, and efficacy, as appropriate. Summary statistics, including median and ranges, are provided for continuous variables. Frequency and percentage are summarized by the treatment cohort for binary and categorical variables.

## 3. Results

### 3.1. Patient Characteristics

Between 6 February 2018 and 9 July 2019, nine patients with pretreated advanced solid tumors were enrolled in this clinical trial and initiated study treatment.

Seven patients (78%) were female and the median age was 55 years. Seven patients (78%) had an ECOG performance status of 1 at the time of enrolment. After careful consideration, two patients with an ECOG performance status of 2 were given a waiver for study participation. In this trial, four patients with cutaneous melanoma, three patients with triple-negative breast cancer, one patient with serous ovarian carcinoma, and one patient with anaplastic thyroid carcinoma were treated.

The patients were heavily pretreated (median of four prior lines of systemic therapy). All four melanoma patients had previously failed treatment with both anti-PD-1 and anti-CTLA-4 monoclonal antibodies, at standard doses, either in monotherapy or in combination therapy. Five patients (56%) had also progressed after prior radiotherapy to lesions that were injected during the trial. Table 1 lists baseline patient and disease characteristics, as well as prior therapies.

### 3.2. MyDC Isolation and Characterization

All of the patients successfully underwent a leukapheresis, followed by a BDCA-1^+^/CD14^-^ cell isolation procedure. A median number of 21 × 10^6^ CD1c (BDCA-1)^+^ myDCs (range 6 × 10^6^–39 × 10^6^) CD1c (BDCA-1)^+^ myDCs with a median purity of 88% (range 51–93%), and a median cell viability of 97% (range 88–99%) was obtained from the nine study patients. The gating strategy of a representative sample is shown in Figure A1. All of the patients were injected intratumorally with their respective individual total number of isolated CD1c (BDCA-1)^+^ myDCs, and were evaluable for toxicity.

### 3.3. Treatment Disposition

At the first treatment session, a median of 1 (range 1–5) lesion was injected. In patients where more than one lesion was injected, the total amount of CD1c (BDCA-1)^+^ myDCs was distributed between the different lesions that were selected beforehand. One day before the injection of the CD1c (BDCA-1)^+^ myDCs, the same lesions were injected with ipilimumab and avelumab. The total number of intratumoral administrations of ipilimumab and avelumab was a median of 4 (range 2–17) in individual patients. A median of 4 (range 2–11) IV administrations of nivolumab was administered. Table A1 provides a descriptive summary of the treatment disposition per patient.

### 3.4. Safety

The treatment-related adverse events were mainly low-grade, and they were mainly limited to local injection-site reactions. These included pain during intratumoral injection of both drugs and myDCs (G1 in two patients, and G2 in one patient necessitating local anesthesia). Subacute local adverse events at the injection site consisted of G1 redness of the skin overlying the injected lesion (*n* = 2) and G1 local pruritus at the injected lesion (*n* = 2). One patient experienced G1 paresthesia in the area of injected lesions. Forty-four weeks after the initiation of study treatment, one melanoma patient developed a G2 bullous pemphigoid that was located on the limbs and thorax that was reversible after topical corticosteroid application. Two patients developed G1 generalized pruritus. In three patients, treatment interruption and systemic corticosteroid treatment were indicated for G2 and G3 pneumonitis and a G3 colitis. All of the treatment-related adverse events were completely reversible and there were no grade 4 or 5 adverse events. Table 2 lists all treatment-related adverse events.

### 3.5. Clinical Outcome

Seven patients (78%) were evaluable for tumor response by ^18F^FDG-PET/CT after 12 weeks of study treatment. Two patients with triple-negative breast carcinoma who had locoregional recurrent disease that was not measurable on CT-imaging were evaluated on a clinical basis and by ^18F^FDG-PET/CT.

**Figure 1:**
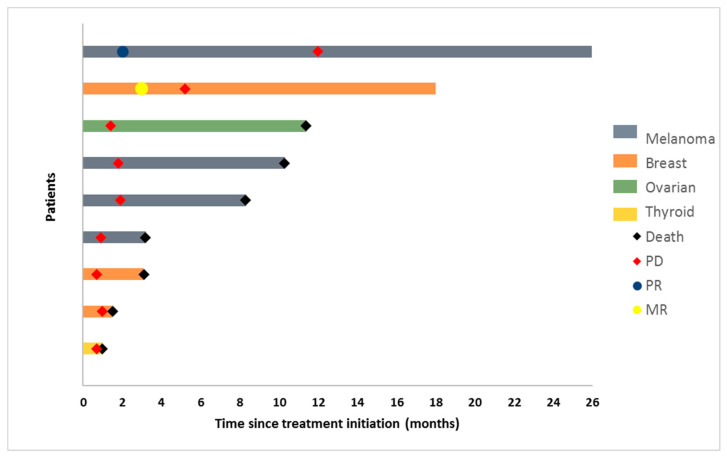
Swimmer plot representing the response kinetics and duration of response of the individual patients according to iRECIST. Each bar represents an individual patient. Patients with melanoma, ovarian cancer, breast cancer, thyroid cancer are depicted by, respectively, a blue, green, orange, or yellow bar. Death, progressive disease, partial response, mixed response are depicted by a black square, a red square, a blue dot, and a yellow dot, respectively.

**Figure 2:**
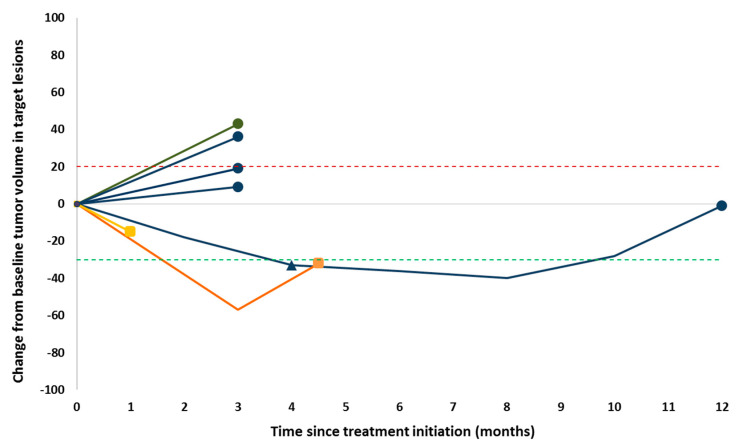
Spider plot representing the change from baseline in tumor burden in patients evaluable with ^18F^FDG-PET/CT-imaging. Two patients initially had only clinically evaluable target lesions which were not measurable on CT, thereby are not depicted in this plot. Patients with melanoma, ovarian cancer, breast cancer, thyroid cancer are depicted by respectively a blue, green, orange, or yellow line. Progressive disease, mixed response, and partial remission are respectively depicted as a dot, square, or triangle respectively at the end of a line.

The best overall response according to iRECIST criteria was a confirmed partial response (PR) in one patient (11%), stable disease (SD) in two (22%) patients (with regression of their injected metastases), and progressive disease (PD) in six (67%) patients (with regression of injected metastases in 3 patients). Figure 1 and Figure 2 depict the best response change from baseline and duration of response per individual patient.

**Figure 3:**
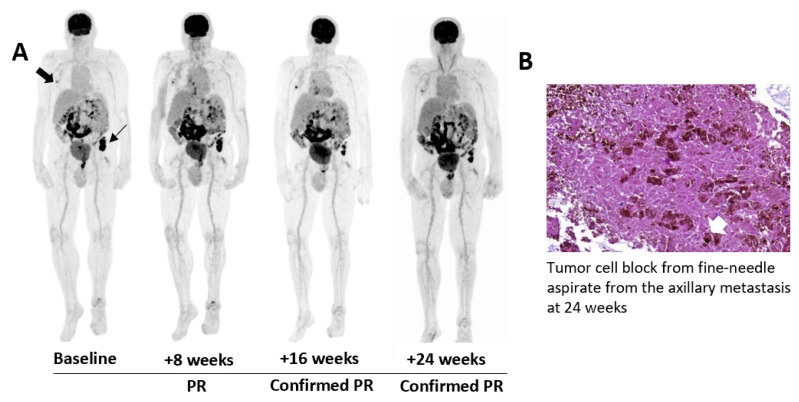

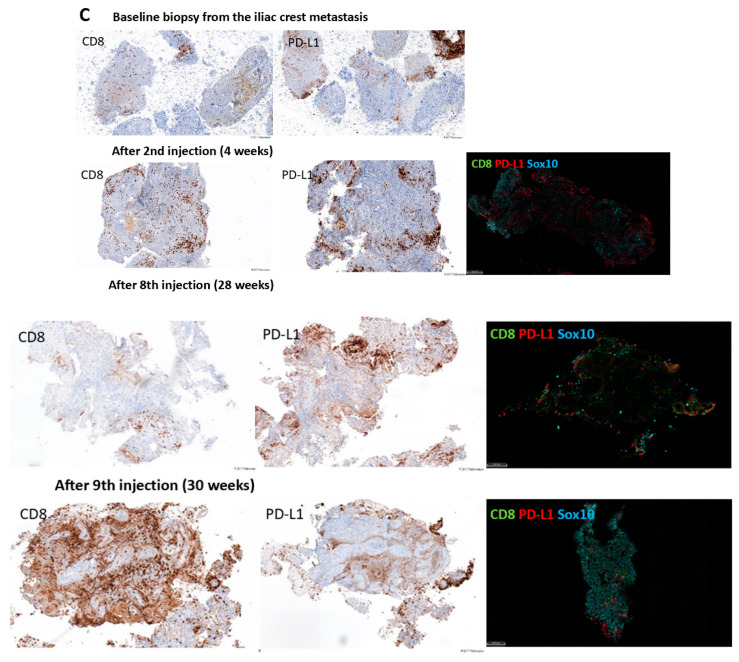
Case illustration of a 72-year-old male patient (myDC-04) with AJCC stage IV-M1d melanoma who achieved a partial response after five intratumoral injections of ipilimumab and avelumab and a single injection of 21 × 10^6^ CD1c (BDCA-1)^+^ myDCs into an axillary lymph node metastasis. (**A**) Tumor response assessment by ^18F^FDG-PET/CT-imaging at baseline, +8 weeks, +16 weeks and +24 weeks. The thick black arrow indicates the only lesion that was injected with CD1c (BDCA-1)^+^ myDCs on day 2 of the treatment schedule. The thin black arrow indicates a lesion that was injected with ipilimumab and avelumab. (**B**) Representative image showing a haematoxylin and eosin stain of a tumor block made from a fine needle aspirate (24 weeks) of the injected axillary lesion. On this section no malignant cells, but pigment incontinence and some melanophages (white arrow) are visible. (**C**) Immunohistochemical and multiplexed immunofluorescent analysis of representative tissue biopsies of the injected iliac crest metastasis at baseline (no multiplexed immunofluorescence due to unavailable tissue), four weeks, 28 weeks, and 30 weeks. Images in the left column show tissue stained for CD8; images in the middle column show tissue stained for PD-L1 (at 200 µm). Markers for multiplexed immunofluorescence included SOX-10, CD8, and PD-L1 (at 200 µm).

One patient with stage IV-M1d melanoma who previously progressed on anti-PD-1 and anti-CTLA-4 checkpoint blockade achieved a durable PR according to iRECIST criteria (Figure 3A, patient myDC-04 in Table A1). At 24 weeks, the axillary node that was initially injected with CD1c (BDCA-1)^+^ myDCs remained slightly metabolically active. Therefore, we performed a FNA which showed no evidence of melanoma cells, but presence of multiple melanophages and remaining melanin pigment (Figure 3B). We resumed intratumoral administrations of ipilimumab and avelumab in the iliac crest metastasis and performed repetitive on-treatment biopsies before every study drug administration. We observed an increase in tumor-infiltrating CD8^+^ T lymphocytes as well as an upregulation of PD-L1. At baseline, rare scattered CD8^+^ lymphocytes were present in the periphery of the lesion. After two intratumoral injections (four weeks after baseline sample) CD8^+^ T lymphocytes were still mainly in the periphery. In subsequent biopsies, an increased infiltration of CD8^+^ T lymphocytes into the centre of the tissue could be observed, with approximately 3% and 15% of infiltrating CD8^+^ T lymphocytes after 28 and 30 weeks, respectively (Figure 3C). PD-L1 expression on IHC increased from 1% expression in the baseline sample to 10% in the sample that was procured after 30 weeks. On-treatment tissue biopsies of injected lesions were not feasible in other patients.

In two additional melanoma patients (myDC-02 and -03), we observed the regression of the injected metastases; nevertheless, non-injected lesions did not respond, which resulted in overall progressive disease. Two patients, one with anaplastic thyroid carcinoma and one with triple-negative breast carcinoma (myDC-05 and -06), achieved a mixed response with PR or SD in injected lesions, as well as a regression of distant non-injected metastases, but occurrence of one or more new lesions. A patient with a recurrent triple-negative breast carcinoma (myDC-09) had a decrease in target lesions; nevertheless, she developed two new bone metastases in vertebrae T6 and T8 as well as a new mediastinal lymph node metastasis. While continuing study treatment, the progressive bone lesions were treated by radiation therapy. Six weeks later, we observed a decreased metabolism of the irradiated vertebral lesions, while the non-irradiated mediastinal lesion had also normalized, but three new bone lesions had appeared. Study treatment was therefore stopped. Table A1 lists an overview of clinical outcome per patient.

## 4. Discussion

In this first-in-human exploratory phase IB clinical trial intratumoral co-injection of CD1c (BDCA-1)^+^ myDCs isolated from the peripheral blood, ipilimumab, and avelumab plus intravenous low-dose nivolumab was feasible and safe in patients with advanced solid tumors. Moreover, early indications were found that the combination of myDCs and immune checkpoint inhibition may be active in patients who previously progressed on immune checkpoint inhibitors or are affected by tumor types that are known to be largely resistant to immune checkpoint inhibitors treatment. Interestingly, one patient with metastatic melanoma who previously progressed on anti-PD-1 and anti-CTLA-4 checkpoint blockade achieved a fast and durable partial response. Histopathological examination of on-treatment tumor biopsies in this patient suggests that the experimental therapy succeeded in stimulating the infiltration of T lymphocytes and, thereby, restoring an effective cancer-immunity cycle that was also effective against non-injected metastases. While regression or stabilization was documented for most of the injected metastases, most patients progressed at pre-existing non-injected metastases and/or developed new lesions on therapy. This suggests that the experimental therapy was insufficiently capable of generating an effective antitumor immune response or that local immune-evasive mechanisms withheld such a response from eradicating non-injected metastases. The antigen-agnostic approach of our experimental therapy would have necessitated a wide-screen for treatment-induced adaptive immune responses, an endeavor that was beyond the objectives of this first-in-human exploratory trial.

Noteworthy advances in cancer immunotherapy were achieved by blocking the PD-1/PD-L1/-L2 immune checkpoint axis, which is believed to rely on the pre-existence of an effective cancer-immunity cycle in which the eradication of the tumor cells is solely restricted by this inhibitory immune checkpoint signaling [41]. Therefore, the approach of cancer immunotherapy has been rather T-cell centered while underestimating the myeloid compartment as playing a possible role in serving as a target for treatment possibilities. It has been suggested that the migration of myDCs from the blood to the TME and subsequent maturation and trafficking to lymphoid structures are likely to be defective or absent in immune checkpoint inhibitor refractory solid tumors; therefore, we sought to find evidence for this by reconstituting the presence of CD1c (BDCA-1)^+^ myDCs isolated from the blood through intratumoral injection. In order to increase the amount of available tumor antigen and optimize the maturation potential of CD1c (BDCA-1)^+^ myDCs in situ, the IgG_1_ subtype monoclonal antibodies ipilimumab and avelumab were co-injected in order to induce ADCC and CDC.

Our first attempt to clinically validate the hypothesis that reconstitution of the TME with unmanipulated CD1c (BDCA-1)^+^ myDC isolated from the blood proved that this is a feasible approach. However, beyond the utility of using CD1c (BDCA-1)^+^ myDC, CD141 (BDCA-3)^+^ myDC would also be of interest, because this subtype of myDC is likely more potent in cross-presenting tumor antigens and stimulating CD8^+^ CTL responses, as compared to CD1c (BDCA-1)^+^ myDC that are suspected to mainly stimulate CD4^+^ T-cell responses. However, the clinical grade isolation of BDCA-1^+^ myDC was not yet available at the time this trial was initiated.

The mode of action of CTLA-4 blockade in humans has not been elucidated completely, but it is likely to involve an expansion of the T-cell repertoire, most likely dependent on an intact process of tumor antigen cross-presentation in secondary and tertiary lymphoid structures. For both of these established immune checkpoint therapies to be successful, there needs to be an initial phase of immune recognition of the cancer cells and subsequently antitumor T cells need to be able to gain access and exert their function within the TME. By their IgG_1_ nature, the monoclonal antibody ipilimumab may be capable of eliciting ADCC and CDC against CTLA-4 expressing cells (including regulatory T cells). Evidence for such a mechanism of action has been found in animal models, but not in humans treated systemically [42]. While higher systemic doses of ipilimumab result in higher overall response rates (ORR) and survival, systemic dosing of ipilimumab is limited by its dose-dependent toxicity [43,44]. Speculative, higher intratumoral doses of ipilimumab may achieve different effects in the TME as compared to IV dosing. Dendritic cell-based vaccines, predominantly with *ex vivo*-cultured monocyte-derived DC (moDC), have been shown to be safe and capable of inducing antitumoral activity in various solid tumors, even in patients with advanced disease; however, clinical responses were variable. Due to higher expression of major histocompatibility complex and functional specialization, natural-occurring DCs, such as myDCs, possess higher antigen-presenting capacities when compared to moDCs [25].

We used a low dose of IV nivolumab (10 mg every two weeks, substantially lower than standard dosing) in this trial to complement the intratumoral injections. In a prospective phase II trial in melanoma in the adjuvant setting, the same low-dose of nivolumab, with or without a single low-dose of ipilimumab, was associated with a 50% incidence of immune-related adverse events (irAE) (all grades), indicating a biologically active regimen [45]. Furthermore, in a phase I trial with a dose escalation of nivolumab in advanced melanoma patients, a nivolumab dose of 0,1 mg/kg showed ORR and irAE that were comparable to higher doses [46]. Pharmacokinetic investigations indicated durable PD-1 receptor occupancy on circulating T cells with ranging doses of nivolumab [47].

Our clinical trial is the first trial worldwide that investigates the intratumoral administration of natural occurring myDCs without any substantial ex vivo manipulation. Naturally occurring DCs, both myDCs and plasmacytoid DCs (pDCs) alone or in combination, have been, or are currently, under investigation as a “classical” DC vaccine in several studies in patients with either prostate cancer or advanced melanoma [25]. Some of these trials are completed and they show encouraging clinical effects, e.g., vaccination with primary myDCs in melanoma patients or vaccination with the combination with myDCs and pDCs in patients with prostate cancer [32,33]. It has also been shown that using pDCs in melanoma patients can result in antigen-specific T-cell responses [31]. These DC vaccines have been shown to be safe and induce antitumoral effects. However, our intratumoral “antigen-agnostic” approach may also be advantageous as it is exploiting the full antigenic potential of the tumor, which includes generating immune responses against private neoantigens, which are believed to be important drivers of immune rejection in solid tumors. Moreover, the preparation of DC vaccines comprises a complex process of ex vivo conditions as well as the necessity of an advanced therapy medicinal product (ATMP)-facility implicating specific infrastructure, more human resources, and much higher production costs. In contrast, when intratumorally injected, the use of autologous, non-substantially manipulated CD1c (BDCA-1)^+^ myDCs has been classified as a non-ATMP by the Committee for Advanced Therapies (EMA). Consequently, a combined immunotherapeutic approach, including the intratumoral administration of myDCs, could be implemented in institutions with more limited infrastructure.

Although this trial has shown promising antitumor responses, there are certain limitations. We should regard the observed clinical effects with precaution, as our study is a phase I clinical trial. Only patients with (sub-)cutaneous, lymph node or other soft tissue metastases were treated in this trial. For safety reasons, patients having only visceral metastases were excluded from this trial, eventually, such patients could potentially be included when efficacy justifies the risk of injecting visceral metastases. Another limitation of this trial is the unavailability of a validated clinical biomarker for treatment response to immune checkpoint inhibition. However, on-treatment biopsies evaluating the dynamics and nature of immune cell infiltration could serve as a “local biomarker”.

Our early findings and early signs of durable antitumoral efficacy legitimate the further exploration of the potential of using intratumoral myDCs in conjunction with other antitumor immunotherapies. Beyond the CD1c (BDCA-1)^+^ myDCs, CD141 (BDCA-3)^+^ myDCs would also be of great interest to be included in the cell product.

## 5. Conclusions

In conclusion, intratumoral injection of autologous, non-substantially manipulated CD1c (BDCA-1)^+^ myDCs with intratumoral co-injection of ipilimumab and avelumab is feasible and safe. Treatment-related adverse events are mainly low-grade and manageable. This treatment regimen resulted in encouraging early signs of antitumoral activity in pretreated patients. Therefore, we think that our therapeutic approach of using intratumoral injection of myDCs that were isolated from the blood deserves further evaluation.

## Figures and Tables

**Table 1:** Baseline patient and disease characteristics.

Characteristics		Patients N (%)
Age (in years)	Median Range	55 40–72
Sex	Male Female	2 (22) 7 (78)
ECOG performance status	0	0
	1	7 (78)
	2	2 (22)
Prior lines of systemic therapy	Median	4
	1–3	4 (44)
	4–6	2 (22)
	7–10	3 (34)
Prior types of systemic therapy	Targeted therapy *	5 (56)
	Chemotherapy	7 (78)
	Immunotherapy	4 (44)
Primary tumor type	Cutaneous melanoma	4 (44)
	Triple negative breast carcinoma	3 (34)
	Serous ovarian carcinoma	1 (11)
	Anaplastic thyroid carcinoma	1 (11)
Prior irradiation of injected lesion	Yes	5 (56)
	No	4 (44)

Targeted therapy (*) includes dabrafinib/trametinib (BRAF-/MEK-inhibition) in melanoma, olaparib (poly ADP ribose polymerase-inhibitor) in ovarian carcinoma. ECOG: Eastern Cooperative Oncology Group.

**Table 2:** Treatment-related adverse events according to CTCAE 5.0 (*n* = 9).

Adverse Event	All Grades N (*%)*	Grade 1–2 N (%)	Grade 3 N (%)	Grade 4 N (%)
Pruritus (generalised)	3 (33)	3 (33)	0	0
Pruritus (local)	3 (33)	3 (33)	0	0
Fatigue	2 (22)	2 (22)	0	0
Injection-site pain	2 (22)	2 (22)	0	0
Paresthesia	2 (22)	2 (22)	0	0
Pneumonitis	2 (22) *	2 (22)	0	0
Redness at injection-site	2 (22)	2 (22)	0	0
Bullous pemphigoid	1 (11)	1 (11)	0	0
Colitis	1 (11) *	0	1 (11)	0
Rash	1 (11)	1 (11)	0	0
Flu-like symptoms	1 (11)	1 (11)	0	0
Hypokalemia	1 (11)	0	1 (11)	0

* These patients received cortisone treatment. *CTCAEv5.0*: Common Terminology Criteria for Adverse Events.

## Appendix A

**Table A1:** Treatment disposition & clinical outcome.

Study Patient	Primary Tumor Type	Type of Injected lesion(s)	N° Baseline Lesions Injected	N° IT Injections	N° IV nivolumab	Response in Injected Lesion(s)	Overall Response
myDC-01	Ovarian	LN	1	6	8	PD	PD
myDC-02	Melanoma	SC	1	4	4	PR	PD
myDC-03	Melanoma	LN	1	7	7	PR	PD
myDC-04	Melanoma	LN and ST	2	17	5	PR	PR
myDC-05	Breast	LN	2	6	11	PR	Mixed response
myDC-06	Thyroid	ST	1	4	2	SD	Mixed response
myDC-07	Breast	C	1	2	3	PD	PD
myDC-08	Melanoma	C	5	3	3	PD	PD
myDC-09	Breast	C	3	3	3	PR	PD

SC: subcutaneous LN: lymph nodes ST: soft tissue C: cutaneous PD: progressive disease, SD: stable disease CR: complete response PR: partial response.
